# Supplementary material for: Addressing the dichotomy of fishing and climate in fishery management with the FishClim model
Source: Commun Biol. 2022 Nov 9;5:1146. doi: 10.1038/s42003-022-04100-6 (PMC9646776; doi:10.1038/s42003-022-04100-6)

**Supplementary Materials**

# Addressing the dichotomy of fishing and climate in fishery management with the FishClim model

Grégory Beaugrand[1*], Alexis Balembois[1], Loïck Kléparski[1,3], Richard R Kirby[4,5]

[1] Univ. Littoral Côte d'Opale, CNRS, Univ. Lille, UMR 8187 LOG, F-62930 Wimereux, France

[3] Marine Biological Association, Citadel Hill, Plymouth PL1 2PB, United Kingdom

[4] The Secchi Disk Foundation, Kiln Cottage, Gnaton, Yealmpton, Devon PL8 2HU, United Kingdom

[5] Ronin Institute, Montclair, NJ 07043, USA

*Corresponding author: gregory.beaugrand@univ-lille.fr

## Supplementary Text 1. Climate projection data

**CNRM-ESM2-1:**

Seferian, Roland (2018). *CNRM-CERFACS CNRM-ESM2-1 model output prepared for CMIP6 CMIP historical*. Version 2018-07-14. Earth System Grid Federation. https://doi.org/10.22033/ESGF/CMIP6.4068

Voldoire, Aurore (2019). *CNRM-CERFACS CNRM-ESM2-1 model output prepared for CMIP6 ScenarioMIP ssp245*. Version 2018-10-26. Earth System Grid Federation. https://doi.org/10.22033/ESGF/CMIP6.4191

Voldoire, Aurore (2019). *CNRM-CERFACS CNRM-ESM2-1 model output prepared for CMIP6 ScenarioMIP ssp585*. Version 2019-09-24. Earth System Grid Federation. https://doi.org/10.22033/ESGF/CMIP6.4226

**GFDL-ESM4:**

Krasting, John P.; John, Jasmin G; Blanton, Chris; McHugh, Colleen; Nikonov, Serguei; Radhakrishnan, Aparna; Rand, Kristopher; Zadeh, Niki T.; Balaji, V; Durachta, Jeff; Dupuis, Christopher; Menzel, Raymond; Robinson, Thomas; Underwood, Seth; Vahlenkamp, Hans; Dunne, Krista A.; Gauthier, Paul PG; Ginoux, Paul; Griffies, Stephen M.; Hallberg, Robert; Harrison, Matthew; Hurlin, William; Malyshev, Sergey; Naik, Vaishali; Paulot, Fabien; Paynter, David J; Ploshay, Jeffrey; Reichl, Brandon G; Schwarzkopf, Daniel M; Seman, Charles J; Silvers, Levi; Wyman, Bruce; Zeng, Yujin; Adcroft, Alistair; Dunne, John P.; Dussin, Raphael; Guo, Huan; He, Jian; Held, Isaac M; Horowitz, Larry W.; Lin, Pu; Milly, P.C.D; Shevliakova, Elena; Stock, Charles; Winton, Michael; Wittenberg, Andrew T.; Xie, Yuanyu; Zhao,

Ming (2018). *NOAA-GFDL GFDL-ESM4 model output prepared for CMIP6 CMIP historical*. Version 2019-08-03. Earth System Grid Federation. https://doi.org/10.22033/ESGF/CMIP6.8597

John, Jasmin G; Blanton, Chris; McHugh, Colleen; Radhakrishnan, Aparna; Rand, Kristopher; Vahlenkamp, Hans; Wilson, Chandin; Zadeh, Niki T.; Dunne, John P.; Dussin, Raphael; Horowitz, Larry W.; Krasting, John P.; Lin, Pu; Malyshev, Sergey; Naik, Vaishali; Ploshay, Jeffrey; Shevliakova, Elena; Silvers, Levi; Stock, Charles; Winton, Michael; Zeng, Yujin (2018). *NOAA-GFDL GFDL-ESM4 model output prepared for CMIP6 ScenarioMIP ssp245*. Version 2019-06-17. Earth System Grid Federation. https://doi.org/10.22033/ESGF/CMIP6.8686

John, Jasmin G; Blanton, Chris; McHugh, Colleen; Radhakrishnan, Aparna; Rand, Kristopher; Vahlenkamp, Hans; Wilson, Chandin; Zadeh, Niki T.; Dunne, John P.; Dussin, Raphael; Horowitz, Larry W.; Krasting, John P.; Lin, Pu; Malyshev, Sergey; Naik, Vaishali; Ploshay, Jeffrey; Shevliakova, Elena; Silvers, Levi; Stock, Charles; Winton, Michael; Zeng, Yujin (2018). *NOAA-GFDL GFDL-ESM4 model output prepared for CMIP6 ScenarioMIP ssp585*. Version 2019-06-18. Earth System Grid Federation. https://doi.org/10.22033/ESGF/CMIP6.8706

**IPSL-CM6A-LR:**

Boucher, Olivier; Denvil, Sébastien; Levavasseur, Guillaume; Cozic, Anne; Caubel, Arnaud; Foujols, Marie-Alice; Meurdesoif, Yann; Cadule, Patricia; Devilliers, Marion; Ghattas, Josefine; Lebas, Nicolas; Lurton, Thibaut; Mellul, Lidia; Musat, Ionela; Mignot, Juliette; Cheruy, Frédérique (2018). *IPSL IPSL-CM6A-LR model output prepared for CMIP6 CMIP historical.* Version 2018-07-11. Earth System Grid Federation. https://doi.org/10.22033/ESGF/CMIP6.5195

Boucher, Olivier; Denvil, Sébastien; Levavasseur, Guillaume; Cozic, Anne; Caubel, Arnaud; Foujols, Marie-Alice; Meurdesoif, Yann; Cadule, Patricia; Devilliers, Marion; Dupont, Eliott; Lurton, Thibaut (2019). *IPSL IPSL-CM6A-LR model output prepared for CMIP6 ScenarioMIP ssp245*. Version 2018-12-05. Earth System Grid Federation. https://doi.org/10.22033/ESGF/CMIP6.5264

Boucher, Olivier; Denvil, Sébastien; Levavasseur, Guillaume; Cozic, Anne; Caubel, Arnaud; Foujols, Marie-Alice; Meurdesoif, Yann; Cadule, Patricia; Devilliers, Marion; Dupont, Eliott; Lurton, Thibaut (2019). *IPSL IPSL-CM6A-LR model output prepared for CMIP6 ScenarioMIP ssp585.* Version 2018-12-18. Earth System Grid Federation. https://doi.org/10.22033/ESGF/CMIP6.5271

**UKESM1-0-LL:**

Tang, Yongming; Rumbold, Steve; Ellis, Rich; Kelley, Douglas; Mulcahy, Jane; Sellar, Alistair; Walton, Jeremy; Jones, Colin (2019). *MOHC UKESM1.0-LL model output prepared for CMIP6 CMIP historical.* Version 2019-11-04. Earth System Grid Federation. https://doi.org/10.22033/ESGF/CMIP6.6113

Good, Peter; Sellar, Alistair; Tang, Yongming; Rumbold, Steve; Ellis, Rich; Kelley, Douglas; Kuhlbrodt, Till (2019). *MOHC UKESM1.0-LL model output prepared for CMIP6 ScenarioMIP ssp245.* Version 2019-11-04. Earth System Grid Federation. https://doi.org/10.22033/ESGF/CMIP6.6339

Good, Peter; Sellar, Alistair; Tang, Yongming; Rumbold, Steve; Ellis, Rich; Kelley, Douglas; Kuhlbrodt, Till (2019). *MOHC UKESM1.0-LL model output prepared for CMIP6 ScenarioMIP ssp585*. Version 2019-11-04. Earth System Grid Federation. https://doi.org/10.22033/ESGF/CMIP6.6405

**Supplementary Figures**

**Supplementary Figure 1.** Shape of the different types of niches used in our model to assess maximum standardised SSB (i.e. K). **a.** Gaussian asymmetrical thermal niche. **b.** Trapezoidal asymmetrical bathymetric niche. **c.** Rectangular trophic niche with a threshold of chlorophyll higher or equal to 0.05 $mg.m^{-3}$. Then an average of the number of days above the threshold was calculated 15 days prior to the target day for each day of the period 1850-2100, or 1850-2300 in the case of the IPSL ESM for scenario SSP 585. The three niches were then combined together by multiplying them at a daily scale. Then an annual average was calculated by using the time period March to October, which is a key period for marine production in the North Sea [90].

**Supplementary Figure 2**. Histogram of the number of geographical cells with a cod occurrence as a function of sea surface temperature (blue bar) from Beaugrand and colleagues[31] and the thermal response curve chosen in this study (red).

**Supplementary Figure 3.** Procedure used to determine the standardisation of ICES SSB. Standardisation of ICES Spawning Stock Biomass (SSB) should be at or below any point of the maximum dSSB (blue line). A number of standardisations was attempted (black thin curves) and we retained the one (red thick curve) that maximised correlation between fishing intensity α and ICES fishing effort F (see Fig 1.e).

**Supplementary Figure 1**

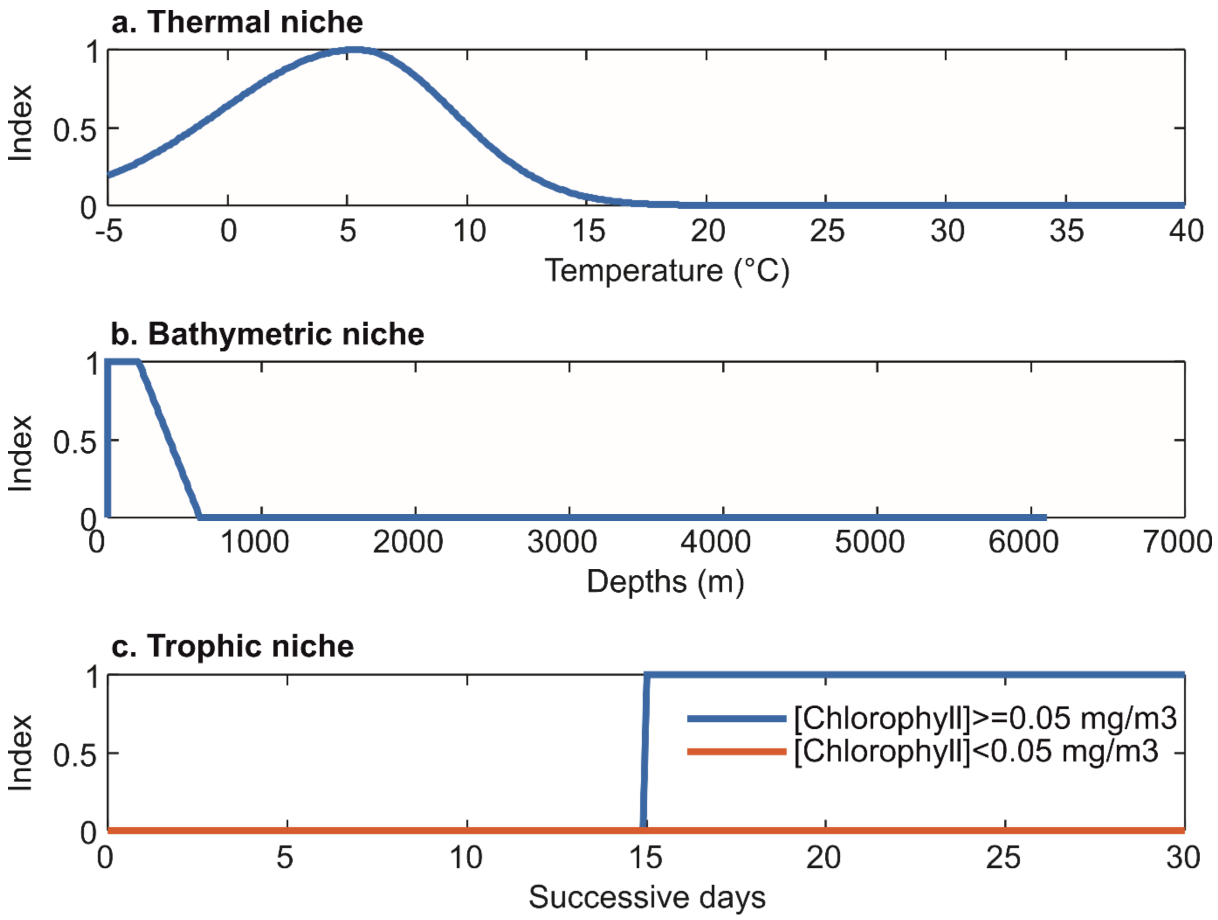

**Supplementary Figure 2**

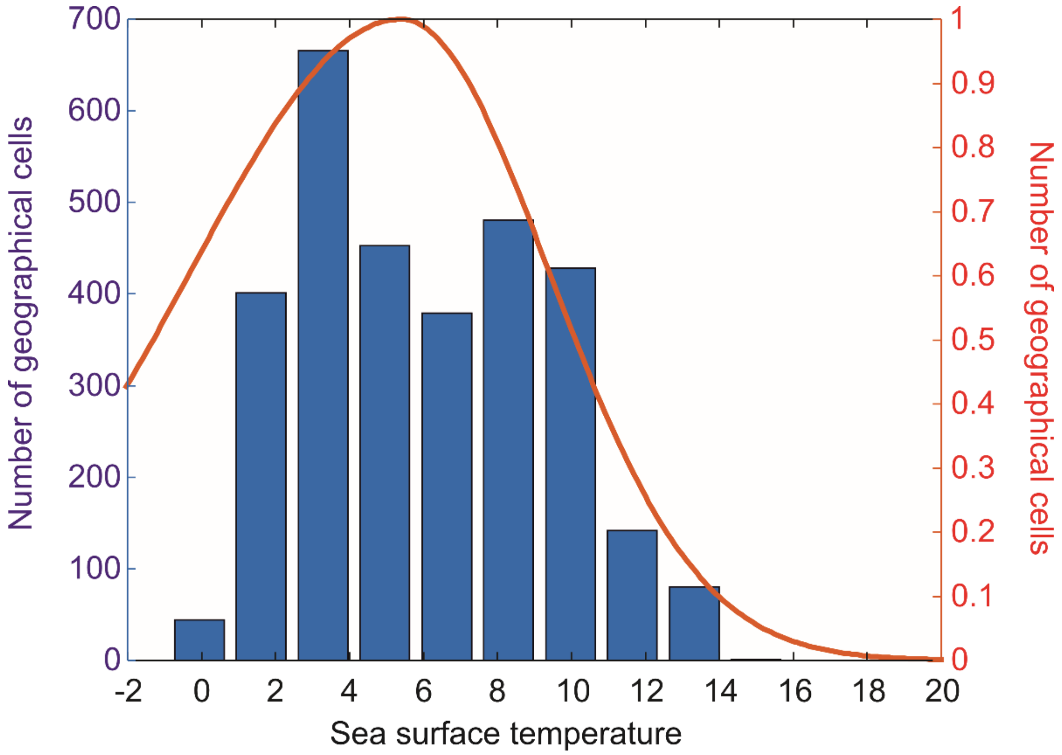

**Supplementary Figure 3**

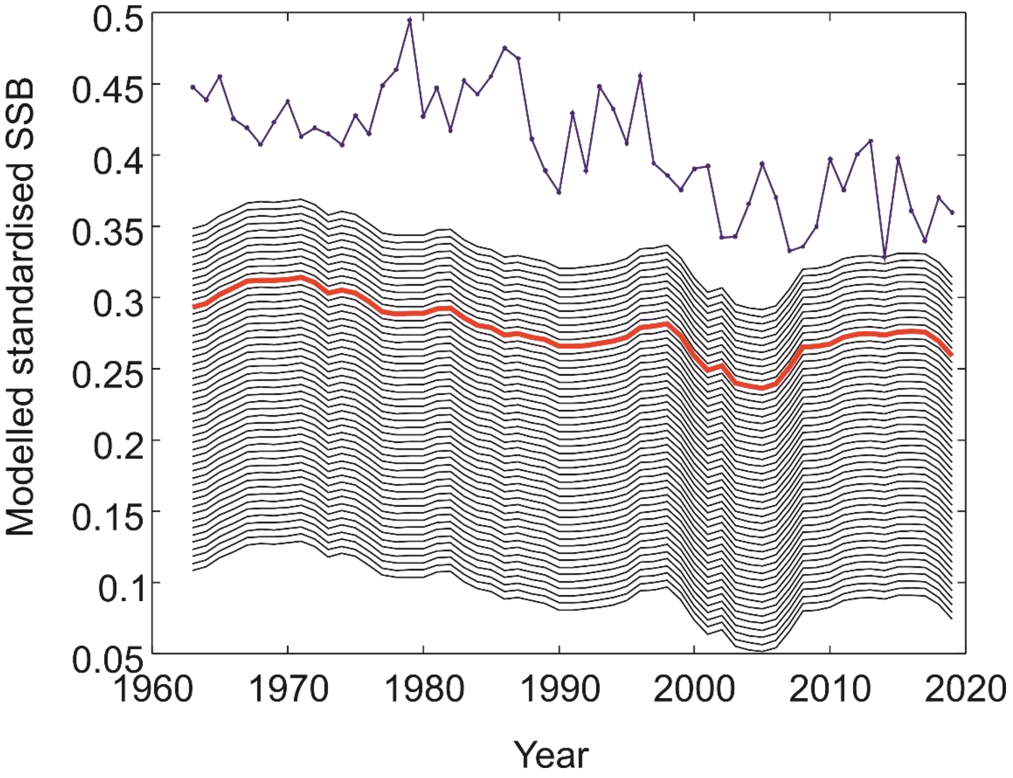

Supplement: Supplementary file 2 — Supplementary Information-New [file 42003_2022_4100_MOESM2_ESM.pdf]
