# Supplementary material for: Addressing the dichotomy of fishing and climate in fishery management with the FishClim model
Source: Commun Biol. 2022 Nov 9;5:1146. doi: 10.1038/s42003-022-04100-6 (PMC9646776; doi:10.1038/s42003-022-04100-6)
Supplement: Supplementary file 3 — Description of Additional Supplementary Data [file 42003_2022_4100_MOESM3_ESM.pdf]

Description of Additional Supplementary Files

File name: Supplementary Data

Description: The source data behind the graphs in the paper
